# Practical Aspects of Twin Pregnancy Diagnosis in Cattle

**DOI:** 10.3390/ani11041061

**Published:** 2021-04-08

**Authors:** Zoltán Szelényi, Ottó Szenci, Levente Kovács, Irina Garcia-Ispierto

**Affiliations:** 1 Department of Obstetrics and Food Animal Medicine Clinic, University of Veterinary Medicine, 1078 Budapest, Hungary; Szenci.Otto@univet.hu; 2 Institute of Animal Sciences, Hungarian University of Agriculture and Life Sciences, 7400 Kaposvár, Hungary; Kovacs.Levente@uni-mate.hu; 3 Agrotecnio Center, Animal Science Department ETSEA, University of Lleida, 25198 Lleida, Spain; Irina.Garcia@udl.cat

**Keywords:** cattle, PAG, pregnancy loss, PSP-B, rectal palpation, twin, ultrasound

## Abstract

**Simple Summary:**

This review summarizes the clinical background, possibilities, and limitations of twin pregnancy diagnosis in cattle, with a special emphasis on pregnancy loss aspects. Due to the fact that reproductive performance is strongly affected by twin pregnancy, clinical veterinarians should become familiar with the correct diagnosis of this phenomenon. Thus, each herd must plan a herd-specific management practice to detect twin pregnancies. A highly accurate diagnostic tool is required to detect such pregnancies, already during the first month of gestation. This commentary review focuses on the diagnostic possibilities and limitations on the field.

**Abstract:**

Twin pregnancies are an economically unwanted phenomenon in dairy cattle, not only because they increase pregnancy losses, but also because antibiotics usage and culling rate of the dam are also dramatically increased due to them, furthermore animal welfare issues are also affected through them. In cattle, under field conditions using an early pregnancy determination tool, the first accurate diagnosis from the pregnancy status is available from around day 28, although further confirmations of pregnancy are required. Twin pregnancy diagnosis is available either by rectal palpation or ultrasonography. The measurement of pregnancy specific proteins are also available to determine gestation, but there is still a long way to go to properly identify twin pregnancies. In this commentary, we compared our own results with the literature data in this field with a special emphasis on the clinical practices.

## 1. Introduction

The diagnosis of twin pregnancy is one of the key factors for reproductive performance in well managed dairy herds. Among the undesirable consequences are higher percentage of pregnancy losses [1,2,3,4], shorter duration of gestation, increased stillbirth and dystocia rates [5,6,7], and increased frequency of postpartum complications (retained placenta, metritis), and consequently the increase of the use of antibiotics [6,8] should be mentioned. Twin pregnancy also has negative consequences on the newborn calves (decreased birth weight, increased mortality) [8]. Another disadvantage is freemartinism [9], due to sex chromosome chimerism resulting in grossly abnormal internal genitalia of freemartin heifers [8,10]. Twin pregnancy also shortens the length of gestation [8,11].

Earlier observational data in Hungary including more than 13,000 calvings in five years reported a 3.4% incidence rate of twin pregnancies [12], with a maximum of eight to nine percent in some years [7,12] at calving time. A much higher twin pregnancy rate can be found at the time of pregnancy diagnosis. A study from Spain reported 20% twin pregnancy rate at the time of ultrasound pregnancy diagnosis, at the end of the first month of gestation [13]. The same study reported an increased frequency of unilateral twins. However, our own data [14] showed an almost equal distribution of unilateral and bilateral twins.

A recent Hungarian study [15] highlighted that about 70% of the dairy farms introduced some technological tool to detect early pregnancy. Another study from the United Kingdom reported more common application [16] and highlighted the importance of evaluation the pregnancy diagnosis. However, in Hungary about one third of the farms still use rectal palpation to detect pregnancy diagnosis. For that reason, nowadays there are no data about regular screening for twin pregnancy.

## 2. Clinical Diagnosis of Twin Pregnancy

During the last four decades, the rate of twin calving has increased [1,17] due to the increase of multiple ovulations associated with high milk yield [13,18] and with the use of different synchronization protocols [2]. Double ovulation occurs more frequently in multiparous cows with high milk production than in primiparous cows [13,18,19].

A recent study advised to set up ‘pivotal periods’, by taking into consideration the practical point of view of the regular reproductive management possibilities. Under field conditions, the application of an early pregnancy determination tool as the first accurate diagnosis is available from around day 28, and further confirmations of pregnancy are required [20]. In our own previous study, pregnancy diagnosis was carried out between days 29 and 42, with an average of day 33.6 [21].

It seems that fulfilment of the metabolic requirements of high-yielding cows stands behind double ovulations (during the first 8 weeks of lactation when milk production peaks). In a study [13], cows above a milk production of 50 kg/day showed a rate of multiple ovulations higher than 50 percent.

### 2.1. Rectal Palpation

Pregnancy diagnosis can be performed in several ways, such as palpation of the amniotic vesicle [22]. The vesicle(s) of about 1–2 cm in diameter can be palpated on the same side where the ovaries carry the corpora lutea. Experienced practitioners can palpate the amniotic vesicle as early as on day 30 of pregnancy. The other possibility for palpating pregnancy is the technique of fetal membrane slipping. It is not commonly used in Europe, while it is more widely applied in the United States [23].

Most of these techniques carry the risk of damaging the amniotic vesicle [24,25,26,27,28]. According to Day et al. [29] and Karlsen et al. [30], rectal manipulation carries the highest risk of damaging the embryo. The general pregnancy loss in the above-mentioned reports varied between 3% and 10%. Romano [23] examined cows in early gestation by ultrasonography, and then also used the fetal membrane slipping technique between days 34 and 41 of pregnancy. It was found that repeated ultrasound examinations—when carrying out the confirmational examination with ultrasound—did not increase the pregnancy loss. When analyzing the data of all cows giving data to the study, the total pregnancy loss ratio was 14%. It was concluded that the increased number of rectal palpations and/or the possible endogenous release of prostaglandins might have contributed to this.

In Hungary, palpation of fluctuation in the pregnant uterine horn is widely used at days 42–48 of pregnancy [15]. Positive pregnancy diagnosis due to the accumulation of allantoic fluid means an enlarged/asymmetric pregnant uterine horn, while experienced practitioners can successfully perform this examination already between days 30 and 40 of gestation [23,31]; however, pathologic conditions such as pyometra and metritis will present the same signs; therefore, this method should never be used as a stand-alone pregnancy diagnosis method [28].

By rectal palpation, bilateral twins can be detected by enlargement of the uterine horns. On the contrary, unilateral twin pregnancies remains unnoticed. For these reasons, rectal palpation cannot be used as a routine screening technique of twin pregnancies.

Concerning twins, a result of the ovulation of co-dominant follicles [30] two corpora lutea (CL) are formed, and two embryos will start to develop in the uterine horn(s). The occurrence of monozygotic twins, originating from a single ovulation and spontaneous division of the embryo, is rare, around 5% among all twins [32]. Triplets or higher-order twins are extremely rare in cattle. Although two CL can be present on the same ovary (meaning usually two embryos in the same uterine horn), bilateral twins are more common than unilateral ones, therefore the presence of two CL not necessarily means twins, while double ovulations are much more common than twin pregnancy [13].

### 2.2. Transrectal Ultrasonography (TRUS)

Ultrasonography was first introduced into bovine reproduction management in the 1980s. Curran et al. [33] determined the times when different fetal organs of the embryo could be detected. In their study, embryonic heartbeat was detectable as early as on days 22–24 post AI. Nowadays, this is the diagnostic criterion for a positive pregnancy diagnosis. It is difficult to evaluate fetal heartbeat at that early stage in practice, therefore the most implemented period to diagnose pregnancy with transrectal ultrasonography is between 28–35 days of gestation. [34,35,36]. The allantoic fluid is first visible from days 25–26 in multiparous cows and from days 23–24 in heifers [23]. Furthermore, the presence of the embryo and the active heartbeat are the main criteria for a positive early pregnancy diagnosis by means of TRUS under practical conditions. As regards the location of the embryos, they can be located either unilaterally or bilaterally, unilateral twins used to have an echogenic line connecting the two embryos, acting as a diagnostic tool [10].

In the case of twin pregnancy, both embryos heartbeats, and the amniotic vesicles should be detected in TRUS examination [37]. In case of doubts in twinning or fetus viability, the evaluation of ovarian structures could be assessed: (1) Number, usually two (30) due to the fact that mostly of twin pregnancies come from the ovulation of co-dominant follicles not from monozygotic twins [38]; (2) Quality of CL, meaning that the echogenicity and the size of the CL, which should be at least 17 mm in the largest diameter [39]. Our data confirmed this [14]; furthermore, we found a low number of twin pregnancies with three corpora lutea. The proportion of cows carrying singleton pregnancies with two corpora lutea was around 10%. With the diagnostic possibilities, it is hard to differentiate between singleton pregnancies and a co-dominant pregnancy or a possible partial embryonic mortality [40].

### 2.3. Examination of Pregnancy Proteins

From day 22 of pregnancy, mononuclear cells originating from the trophoblast migrate into the endometrium. Cell migration originating from the trophoblast can be observed throughout the gestation. During this process, they turn into binucleate and, in some cases, trinucleate cells. Because of this migration, the bovine placenta is called a synepitheliochorial placenta [41,42].

Pregnancy-associated glycoproteins can be differentiated into two subgroups: the PAG-2 subgroup [43] was known to mainly be localized at the fetal-maternal borderline, although some reports recently showed them in the maternal plasma [44,45]. The PAG-1 subgroup is expressed mostly on the bi- and trinucleate cells of the trophoblast, although mononucleate cells also secrete it [46,47]. The measurement of them was used to discriminate singleton and twin pregnancies [48]. Molecular cloning studies showed that the amino acid sequence of PSP-B is homologous to that of PAG1, and they are inactive members of the aspartic acid proteinase family [49]. The isolated preparations differed in carbohydrate and sialic acid content. These characteristics may explain the minor differences in their profile and in their disappearance from the maternal circulation after calving or embryonic mortality [50].

Despite 30 years of intensive research, the clear function of the production of these proteins has remained unknown. The welfare and viability of the fetus, as well as pregnancy loss during gestation, can be monitored by their use [45,48,50]

For more than 20 years, different methods were used for measuring pregnancy proteins: blood sera [51] and milk [52,53]. Serum is the method of election because these proteins have lower concentrations in milk [20]. Besides the biological fluid, numerous factors may affect the concentrations of pregnancy proteins in serum. Yániz et al. [54] showed significant differences in PAG-1 levels between individuals depending on the sire, the number of embryos, and the season.

Several studies showed that in dams carrying two embryos, higher concentrations of PAG-1 could be measured, and with the progress of gestation, the elevation of PAG-1 concentration was also higher than in cows carrying singletons [55,56]. Despite this, PAG-1 cannot identify partial pregnancy loss or embryo reduction [56]. Moreover, the shorter half-life of PAG compared to PSP-B is also remarkable [57]. One study determined that TRUS is needed for twin pregnancy diagnosis [58] because clinically applicable cut-off value for the diagnosis of twin pregnancy could only be reached from day 85 of pregnancy.

## 3. The Effect of Twin Pregnancy on Pregnancy Losses under Practical Conditions

There are several factors influencing the pregnancy loss associated to twin pregnancy. One of the most important factors is the embryo’s location in the uterus. Bilateral twin pregnancies resulted in a lower number of pregnancy losses than unilateral twin pregnancies [56], indicating that possibly the physical extensions of two embryos can induce pregnancy loss. Heat stress effect was studied to influences pregnancy losses of dams pregnant with twins [32], and in another study, heat stress also effected pregnancy loss with the same odds as carrying a twin pregnancy [59]. The method of pregnancy diagnosis can also be a source of pregnancy loss; however a recent study, when veterinary students under training were performing rectal palpation [60], could not confirm this. In case of increased losses after early pregnancy diagnosis, to date there is no data and most of the recent scientific results supported the ineffectiveness of the careful examination.

The primary pregnancy diagnoses must be confirmed later [20,56,61] to achieve reliability and to manage herd health issues. This confirmation is usually performed around day 60 of pregnancy when placentation is completed [24]. Most of the losses in singleton and twin pregnancies usually occur between the time of early pregnancy diagnosis and the confirmation of gestation [20,62]. Twin pregnancy loss also showed a peak in late gestation [14].

## 4. Management of Twin Pregnancies

The individual animal performing of induced reduction in case of twin pregnancy is a described method [63,64]. The manual embryo reduction is considered to be the most economic option to mitigate the negative impacts of twinning [65]; however, practical application is not widespread at the moment. The maintaining strategy is based on medical treatments [66] highlighting that the economic circumstances must be taken into consideration as well. The total herd level termination of twins by using prostaglandin F2alpha is a possible strategy [67], but farmers’ resistance should be noted when performing it under practical circumstances. In dairy herds with low incidence rate of twinning, termination of pregnancy can be an option.

In case of a dairy herd, the early diagnosis of twin pregnancies is the primary goal. Previously, it was shown in two independent studies under practical circumstances [14,68], that the application of different synchronization protocols prior AI decreased the percentage of the occurrence of twinning. The possible explanation was that the stronger regulation of the ovarian cycle was decreasing the possibility of the ovulation of coordinated follicles [67]. An alternative solution at herd level can be the screening for twins at the early diagnosis with a selected diagnostic method, after that the identified twins undergo confirmational diagnosis every second week until day 60 of pregnancy in order to rule out partial or total losses. At the time of the initial twin diagnosis, medical support should be given. It was also demonstrated in clinical practice, that calving time stillbirth events dramatically increase due to twinning [14], therefore careful management in the calving barn is also part of the defense on one hand, with the training of the assistance to handle twin calvings and on the other hand to support the newborn animals.

## 5. Conclusions

Based on the data presented above, nowadays all the pregnancy examination methods seem to be feasible in detecting twin pregnancies in dairy cows. The rectal palpation is able to distinguish between unilateral and bilateral twin pregnancies. Transrectal ultrasonography is the most widespread method under practical circumstances, because both unilateral and bilateral twins can be detected. Moreover, heartbeat (and through this fetal viability) and corpora lutea can be monitored. The measurement of pregnancy-specific proteins from biological fluids has been a promising tool for 20 years, but at the moment, the detection of twins is limited. Further studies should be performed in order to increase the accuracy of this technique.

Pregnancy loss also affects reproductive performance with special regard to twin gestations in cattle. While more authors demonstrated an increased occurrence of losing twin pregnancies, especially in the case of unilateral twins, the distribution of laterality shows high variability, resulting under field circumstances various results.
